# The role of magnesium sulfate in the intensive care unit

**DOI:** 10.17179/excli2017-182

**Published:** 2017-04-05

**Authors:** Yunes Panahi, Mojtaba Mojtahedzadeh, Atabak Najafi, Mohammad Reza Ghaini, Mohammad Abdollahi, Mohammad Sharifzadeh, Arezoo Ahmadi, Seyyed Mahdi Rajaee, Amirhossein Sahebkar

**Affiliations:** 1 Clinical Pharmacy Department, Faculty of Pharmacy, Baqiyatallah University of Medical Sciences, Tehran, Iran; 2 Clinical Pharmacy Department, Faculty of Pharmacy, Tehran University of Medical Sciences, Tehran, Iran; 3 Research Center for Rational Use of Drugs, Tehran University of Medical Sciences, Tehran, Iran; 4 Department of Anesthesiology and Critical Care Medicine, Faculty of Medicine, Sina Hospital, Tehran University of Medical Sciences, Tehran, Iran; 5 Department of Neurosurgery and Neurology, Sina Hospital, Tehran University, Iran; 6 Department of Toxicology and Pharmacology, Faculty of Pharmacy, Tehran University of Medical Sciences, Tehran, Iran; 7 Biotechnology Research Center, Mashhad University of Medical Sciences, Mashhad, Iran

**Keywords:** magnesium sulfate, intensive care unit, neuroprotection, ICU

## Abstract

Magnesium (Mg) has been developed as a drug with various clinical uses. Mg is a key cation in physiological processes, and the homeostasis of this cation is crucial for the normal function of body organs. Magnesium sulfate (MgSO_4_) is a mineral pharmaceutical preparation of magnesium that is used as a neuroprotective agent. One rationale for the frequent use of MgSO_4_ in critical care is the high incidence of hypomagnesaemia in intensive care unit (ICU) patients. Correction of hypomagnesaemia along with the neuroprotective properties of MgSO_4_ has generated a wide application for MgSO_4_ in ICU.

## Introduction

Magnesium (Mg) is one of the most abundant cations in the body, and is also a drug with numerous clinical applications. The body usually contains up to 28 g of Mg (Wacker and Parisi, 1968[161]). Most of the Mg is present as an intracellular cation. Of total Mg in the body, 53 % accumulates in bones, 27 % in muscular tissues, 19 % in soft tissues, 0.5 % in red blood cells, and 0.3 % in blood serum (Facchinetti et al., 1991[40]). Half of this Mg is available as free ion and not bound to albumin or anions (Jahnen-Dechent and Ketteler, 2012[68]). Increase or decrease in serum Mg level is associated with impaired body hemostasis and disorders of different organs (Kingston et al., 1986[80]). Hypomagnesaemia is described as serum Mg levels below 1.7 mg/dL, while hypermagnesaemia occurs when the total serum Mg level is higher than 2.6 mg/dL (Kingston et al., 1986[80]; Soesan et al., 2000[147]).

## Physiological Roles of Mg

Magnesium is a vital element that is directly or indirectly involved in the physiological processes (Aikawa, 1980[5]). Magnesium is an essential co-factor for the enzymatic reactions (Aikawa, 1980[5]). This element is particularly involved in the storage and transfer of the energy (Noronha and Matuschak, 2002[118]; Reinhart, 1988[124]). Also, Mg regulates glycolysis-related enzymes (Fawcett et al., 1999[41]). Mg activates a lot of enzymatic systems that are essentially necessary in the metabolism of energy. Magnesium is a calcium antagonist that acts *via* regulating intracellular calcium availability (Romani, 2011[127]). Calcium metabolism and transportation has crucial roles in cardiac function, muscular contraction, blood pressure regulation and neuronal activity (Akhtar et al., 2011[7]; Noronha and Matuschak, 2002[118]). 

Influx and efflux of Mg plays an important role in different transcellular transports (Kolte et al., 2014[84]). Magnesium deficiency induces a systemic stress to respond during activation of neuroendocrine pathways (Mazur et al., 2007[101]); it has been implicated in the pathophysiology of several diseases and reported to be related to increased mortality in ICU patients (Zafar et al., 2014[172]). A defect in any part of the transcellular transports may lead to different diseases such as pre-eclampsia, Parkinson's disease, atrial fibrillation and anoxic brain injury (Kolte et al., 2014[84]). Magnesium has analgesic properties that are due to N-methyl-D-aspartate (NMDA) receptor blocking action (Akhtar et al., 2011[7]). Other physiological roles of Mg include: (1) establishing the electrical potential across cell membranes; (2) involvement in intermediary metabolism; (3) involvement in protein and nucleic acid synthesis; (4) exerting depressant effect at the synapse as Mg affects channels on the cardiac and smooth muscles; (5) cell cycle regulation; (6) mitochondrial functions control; (7) maintaining stability of cell membranes, and (8) supporting cytoskeletal integrity (Aikawa, 1980[5]; Dubé and Granry, 2003[34]; Fawcett et al., 1999[41]; Golf et al., 1993[46]; Gordon, 1963[50]; Mubagwa et al., 2007[111]; Nadler and Rude, 1995[115]; Simpson and Knox, 2004[142]; Volpe and Vezu, 1993[159]; Wacker and Parisi, 1968[161]; White and Hartzell, 1989[167]).

## Magnesium Deficiency in ICU

One of the key reasons for the wide use of Mg in critical care is the high prevalence of hypomagnesaemia in ICU patients (Noronha and Matuschak, 2002[118]; Tong and Rude, 2005[153]). Around 90 % of ICU patients under surgery and 65 % of ICU patients under drug therapy commonly experience hypomagnesaemia (Koch et al., 2002[82]). Hypomagnesaemia is correlated with poor prognosis and high mortality rate in critically ill patients (Dabbagh et al., 2006[30]).

Noronha and Matuschak in 2009 described major causes of Mg deficiency in ICU patient as: (1) reduction of intestinal absorption of Mg, (2) increased loss of Mgbyrenal route, and (3) compartmental redistribution (Noronha and Matuschak, 2002[118]). The most common gastrointestinal (GI) diseases with Mg loss include intestinal malabsorption syndromes, inadequate Mg intake, re-feeding syndrome, chronic diarrhea, short bowel syndrome, fistulae in the intestinal and biliary system, and acute pancreatitis (Booth et al., 1963[17]; Edmondson et al., 1952[35]; Gordon, 1963[50]; Hall and Joffe, 1973[57]; Martin et al., 2009[99]). Long-term use of Proton Pump Inhibitors (PPIs) has also been reported to block intestinal absorption of Mg. The mechanism of this action is an increase in the intestinal lumen PH that proceeds to the reduction of TRPM 6/7 channel affinity for Mg (Thongon and Krishnamra, 2011[151]; William et al., 2014[170]).

Intravenous Mg supplementation rapidly increases serum Mg level following long-term use of PPIs and subsequent hypomagnesaemia. PPIs affect intestinal epithelial cell locally. Oral Mg is not effective in PPI's induced hypomagnesaemia. Discontinuation of PPI use will resultsin quick normalization of serum Mg levels (Mackay and Bladon, 2010[96]; William and Danziger, 2016[169]).

Renal excretion is an important cause of Mg loss in ICU patients. Interstitial nephropathy, post-obstructive diuresis, acute tubular necrosis (diuretic phase), post-renal transplantation and drug-induced Mg wasting (Aminoglycosides, Amphotericin B, Cisplatin, Colony-stimulating factor therapy, Cyclosporine A, Loop and thiazide diuretics, Pentamidine) are reasons for renal Mg loss (Barton et al., 1984[10], 1987[11]; Hellman et al., 1962[61]; Jones et al., 1966[73]; Kingston et al., 1986[80]; Knochel, 1977[81]; Lim and Jacob, 1972[91]; Martin et al., 2009[99]; Noronha and Matuschak, 2002[118]; Shah et al., 1990[137]; Shah and Kirschenbaum, 1991[138]; von Vigier et al., 2000[160]).

Causes of Mg loss due to redistribution of Mg and endocrine disorders include acute respiratory alkalosis, administration of epinephrine, alcoholic ketoacidosis, blood transfusion, diabetic ketoacidosis, hyperaldosteronism, hyperparathyroidism, hyperthyroidism, hungry bone syndrome, and syndrome of inappropriate antidiuretic hormone (al-Ghamdi et al., 1994[8]; Aziz et al., 1996[9]; England et al., 1992[38]; Martin et al., 2009[99]; McLellan et al., 1984[104]; Shane and Flink, 1991[140]; Whyte et al., 1987[168]). Other causes include cardiopulmonary bypass, hypophosphatemia (chronic alcoholism), hypercalcemia/hypercalciuria, excessive sweating and severe burns (al-Ghamdi et al., 1994[8]; Kingston et al., 1986[80]; Martin et al., 2009[99]; Weglicki and Phillips, 1992[165]). 

## Clinical Manifestations of Hypomagnesaemia in the ICU

The symptoms of hypomagnesaemia start when serum Mg levels fall below 1.2 mg/dL (Kingston et al., 1986[80]). These symptoms affect different body organs and depend on the rate of deficiency of ionized Mg (Brenner and Rector, 1991[19]). However, most cases of hypomagnesaemia in intensive care are asymptomatic (Soesan et al., 2000[147]). Clinical manifestations of hypomagnesaemia in ICU patients include muscle cramps, tremor, weakness, hyperreflexia, positive Trousseau or Chvostek sign, carpopedal spasm, tetany, nystagmus, vertigo, aphasia, hemiparesis, delirium, choreoathetosis, supraventricular arrhythmias, ventricular arrhythmias, torsades de pointes, electrolyte disturbance (hypocalcemia, hypokalemia, or both), hypertension, coronary vasospasms, and bronchial airway constriction. Severe hypomagnesaemia may cause generalized tonic-clonic seizures (Burch and Giles, 1977[22]; Iseri et al., 1989[67]; Ralston et al., 1989[123]; Ryzen et al., 1985[131]; Tzivoni et al., 1988[155]; Wacker, 1962[162]; Watanabe and Dreifus, 1972[164]).

## Magnesium Sulfate in ICU

Numerous roles for magnesium in critical care medicine have been suggested (Noronha and Matuschak, 2002[118]). Deficiency of Mg is common in hospitalized patients, and is frequently reported in admitted ICU patients (Koch et al., 2002[82]; Ryzen et al., 1985[131]). Management of patients in ICU is somehow complicated and depends on the conditions of every patient (Honarmand et al., 2012[63]). It has been suggested to employ an established protocol as a base to define a moderate dose of Mg that is safe over the years in ICU (Hebert et al., 1997[60]). 

In 1906, for the first time, magnesium sulfate (MgSO_4_) was used to prevent eclamptic seizures in Germany (Horn, 1906[64]). Magnesium is replaced intravenously with MgSO_4 _when hypomagnesaemia is severe (Ryzen et al., 1985[131]). MgSO_4_ is the essential preparation of intravenous Mg. Magnesium sulfate, usually known as Epsom salt, is an ordinary mineral pharmaceutical preparation of Mg that is used both externally and internally. Both Mg and sulfate absorb through the skin to recover blood levels (Noronha and Matuschak, 2002[118]; Ignatavicius and Workman, 2015[66]). A number of authors have described Mg as “the forgotten electrolyte” (Elin, 1994[37]; Gonzalez et al., 2013[48]). Hypomagnesaemia is a significant but underdiagnosed electrolyte imbalance (Gonzalez et al., 2013[48]). MgSO_4_ has been used during the 20^th^ century for eclamptic seizures' prevention (Lazard, 1925[89]; Pritchard, 1955[121]), and continues to be used widely. 

Numerous mechanisms of action have been suggested for magnesium including (1) vasodilatory action, (2) blood-brain barrier (BBB) protection, (3) reduction of cerebral edema, and (4) central anticonvulsant action (Aali et al., 2007[1]). 

## Clinical Application of Magnesium Sulfate in ICU

### Acute asthma

Asthma has been described as a chronic inflammatory disorder of the airways with an increase of bronchial responsiveness to a variety of stimuli. It is often reversible, either spontaneously or with treatment (Bateman et al., 2008[12]).

Standard treatments for asthma crisis include bronchodilators (short-acting), agonists of β2-receptors, inhaled ipratropium bromide, corticosteroids, anticholinergic drugs and general managements (Bateman et al., 2008[12]). Researchers have suggested MgSO_4_ as a treatment option for patients who are resistant to standard therapy (Bateman et al., 2008[12]; Gontijo-Amaral et al., 2007[47]; Jones and Goodacre, 2009[74]; Kew et al., 2014[77]). Life-threatening conditions like severe asthma attacks require intensive medical care. The beneficial effects of MgSO_4_ have been shown in children and adult patients with severe asthma in the ICU (Boonyavorakul et al., 2000[16]; Daengsuwan and Watanatham, 2016[31]; Griffiths and Kew, 2016[52]; Kew et al., 2014[77]; Kokturk et al., 2005[83]; Rowe, 2013[128]; Rowe and Camargo, 2008[129]; Rower et al., 2017[130]; Singh et al., 2008[143]).

Mechanisms of Mg action for the management of severe asthma include: (1) reduction of intracellular calcium level (blockade of calcium entry, calcium release and activation of Na^+^-Ca^2+^ pumps), (2) muscle relaxation (inhibition of myosin and calcium interaction), (3) reduction of inflammatory mediators (inhibition of degranulation of mast cells and T-cells stabilization), (4) depression of the irritability of muscle fibers, and (5) inhibition of prostacyclin and nitric oxide synthesis. These mechanisms lead to a reduction in the severity of asthma (Gontijo-Amaral et al., 2007[47]; Rowe, 2013[128]).

MgSO_4_ is used *via* intravenous and inhalation routes for the management of acute asthma (Shan et al., 2013[139]). Use of MgSO_4_ through intravenous route in adult and children patients improves respiratory function (Boonyavorakul et al., 2000[16]; Daengsuwan and Watanatham, 2016[31]; Griffiths and Kew, 2016[52]; Kew et al., 2014[77]; Kokturk et al., 2005[83]; Rowe, 2013[128]; Rowe and Camargo, 2008[129]; Rower et al., 2017[130]; Singh et al., 2008[143]). In some countries, the intravenous form of MgSO_4_ is broadly used as an adjunctive therapy for severe acute asthma, especially in patients not responding to initial treatments (British Thoracic Society Scottish Intercollegiate Guidelines, 2008[20]; Jones and Goodacre, 2009[74]). Unlike adults, in children MgSO_4_ has a significant effect on hospital admission (Ciarallo et al., 2000[28], 1996[29]; Gurkan et al., 1999[55]; Porter et al., 2001[120]; Scarfone et al., 2000[133]). The impact of MgSO_4_ on forced expiratory volume in 1 second (FEV1) and peak expiratory flow rate (PEFR) were assessed in different clinical trials (Bessmertny et al., 2002[13]; Bloch et al., 1995[15]; Boonyavorakul et al., 2000[16]; Devi et al., 1997[32]; Gallegos-Solorzano et al., 2010[43]; Green and Rothrock, 1992[51]; Hughes et al., 2003[65]; Mahajan et al., 2004[97]; Silverman et al., 2002[141]; Tiffany et al., 1993[152]). In children, brief infusion and maximum weight-based dosage of MgSO_4_ have been suggested for the management of severely ill asthmatic patients in the ICU (Egelund et al., 2013[36]; Liu et al., 2016[94]). Up to 2.5 gram of Mg loading dose with β-agonist and corticosteroid (methylprednisolone, hydrocortisone, and dexamethasone) were reported to be efficacious in the management of asthma (British Thoracic Society Scottish Intercollegiate Guidelines, 2008[20]). Ipratropium, aminophylline, theophylline and ephedrine are additional drugs in the management of acute asthma (Bloch et al., 1995[15]; Devi et al., 1997[32]; Green and Rothrock, 1992[51]; Singh et al., 2008[143]; Tiffany et al., 1993[152]). However, in contrast to intravenous MgSO_4_, the effect of the inhaled form remains controversial. Up to 500 mg MgSO_4_ for each dose of nebulization has been used in several clinical trials (Aggarwal et al., 2006[3]; Ahmed et al., 2013[4]; Bessmertny et al., 2002[13]; Chande and Skoner, 1992[24]; Gallegos-Solorzano et al., 2010[43]; Gandia et al., 2012[44]; Hill et al., 1997[62]; Hughes et al., 2003[65]; Kokturk et al., 2005[83]; Mangat et al., 1998[98]; Nannini and Hofer, 1997[116]; Nannini et al., 2000[117]; Rolla et al., 1987[126]; Zandsteeg et al., 2009[173]). Respiratory functions and hospital admission were assesed in all studies and, similar to intravenous MgSO_4_ therapy, β-agonists and corticosteroids were used in all patients (Aggarwal et al., 2006[3]; Ahmed et al., 2013[4]; Chande and Skoner, 1992[24]; Gandia et al., 2012[44]; Hill et al., 1997[62]; Mangat et al., 1998[98]; Nannini and Hofer, 1997[116]; Nannini et al., 2000[117]; Rolla et al., 1987[126]; Zandsteeg et al., 2009[173]). In one study, nebulized MgSO_4_ was compared to nebulized salbutamol (Mangat et al., 1998[98]). The authors showed that there is no significant difference between the bronchodilatory effect of nebulized MgSO_4_ and salbutamol in the management of acute asthma (Gonzalez et al., 2013[48]). In 2016, Ling and colleagues reported that nebulized MgSO_4_ is not useful to improve pulmonary function or reduce the number of patients admitted to the hospital in adults with acute asthma (Ling et al., 2016[92]). In children, treatment with nebulized magnesium sulfate showed no significant effect on respiratory function or hospital admission and further treatment (Su et al., 2016[149]). Adverse events have been occasionally reported in the clinical trials, but the most common adverse reactions with MgSO_4 _are cardiac arrhythmia, confusion, drowsiness, flushing, hypotension, loss of deep tendon reflexes, muscle weakness, nausea, respiratory depression, thirst, and vomiting. Rarely, administration of MgSO_4 _can lead to cardiac arrest and coma (Martindale and Westcott, 2008[100]). 

### Magnesium sulfate as a neuroprotective agent

MgSO_4_ has been well documented to be beneficial in the management of nervous system injuries especially in the ICU. These injuries include stroke, aneurysmal subarachnoid hemorrhage (ASAH), and traumatic brain injuries (Afshari et al., 2013[2]; Akdemir et al., 2009[6]; Bradford et al., 2013[18]; Chan et al., 2005[23]; Chen et al., 2015[25]; Chen and Carter, 2011[27]; Dabbagh et al., 2006[30]; Dorhout Mees et al., 2012[33]; Friedlich et al., 2009[42]; Gao et al., 2013[45]; Gonzalez-Garcia et al., 2012[49]; Hassan et al., 2012[58]; James et al., 2009[69]; Jiang et al., 2017[71]; Johnson et al., 1993[72]; Kahraman et al., 2003[75]; Kidwell et al., 2009[79]; Kumar et al., 2015[85]; Lamers et al., 2003[87]; Lampl et al., 2001[88]; Mirrahimi et al., 2015[107]; Mousavi et al., 2004[109], 2010[110]; Muir and Lees, 1995[112]; Muir et al., 2004[113]; Muroi et al., 2008[114]; Rahimi-Bashar et al., 2017[122]; Rinosl et al., 2013[125]; Saver et al., 2015[132]; Selvaraj and Syed, 2014[136]; Singh et al., 2012[144]; Sleeswijk et al., 2008[146]; Stippler et al., 2006[148]; van den Bergh et al., 2005[156]; van Norden et al., 2005[157]; Veyna et al., 2002[158]; Wang et al., 2012[163]; Westermaier et al., 2010[166]; Wong et al., 2010[171]; Zafar et al., 2014[172]; Zhao et al., 2016[174]; Zhu et al., 2004[175]). 

#### MgSO_4_ and Aneurysmal Subarachnoid Hemorrhage (ASAH)

Several studies have been performed on the efficacy and dosage of MgSO_4_ in ASAH in the last two decades (Afshari et al., 2013[2]; Akdemir et al., 2009[6]; Bradford et al., 2013[18]; Chen et al., 2015[25]; Chen and Carter, 2011[27]; Dabbagh et al., 2006[30]; Dorhout Mees et al., 2012[33]; Hassan et al., 2012[58]; Jiang et al., 2017[71]; Kahraman et al., 2003[75]; Kidwell et al., 2009[79]; Kumar et al., 2015[85]; Mousavi et al., 2010[110]; Muir and Lees, 1995[112]; Muir et al., 2004[113]; Muroi et al., 2008[114]; Saver et al., 2015[132]; Selvaraj and Syed, 2014[136]; Singh et al., 2012[144]; Sleeswijk et al., 2008[146]; Stippler et al., 2006[148]; van den Bergh et al., 2005[156]; van Norden et al., 2005[157]; Veyna et al., 2002[158]; Wang et al., 2012[163]; Westermaier et al., 2010[166]; Wong et al., 2010[171]; Zafar et al., 2014[172]; Zhao et al., 2016[174]; Zhu et al., 2004[175]). Different doses of MgSO_4_ have been suggested for neuroprotection. Veyna and colleagues used MgSO_4_ in 20 ASAH patients and showed that high dose of Mg is safe and efficient and can maintain serum Mg levels in the range of 4-5.5 mg/dL. Their study was focused on vasospasm, middle cerebral artery (MCA) velocity and Glasgow Outcome Scale (GOS). The findings showed better outcome in patients with ASAH 90 days post-hemorrhage, but they did not find a significant difference in GOS between the control and treatment groups (Veyna et al., 2002[158]). Also, van Norden et al. (2005[157]) showed that treatment with MgSO_4_ at a dose of 64 mmol/day will result in 1-2 mmol/L of serum Mg level without any side effect. Studies by Van der Bergh and colleagues (2005[156]) revealed that MgSO_4_ delays cerebral ischemia. They used Rankin score to measure outcomes in the patients. Stippler reported the efficacy of Mg in the management of SAH and improving the Rankin score. The mechanism of Mg efficacy in SAH was suggested to involve a significant reduction in vasospasm (Stippler et al., 2006[148]). High dose of MgSO_4_ was also suggested to be prophylactic and associated with better outcomes in SAH patients (Muroi et al., 2008[114]). MgSO_4_ can increase ischemic tolerance in the nervous system at the time of hypo-perfusion, attenuate vasospasm and decrease outcomes in patients with ASAH (Bradford et al., 2013[18]; Chen and Carter, 2011[27]; Westermaier et al., 2010[166]). Despite these findings on the beneficial role of MgSO_4_ in ASAH, in three studies authors did not suggest this drug for ASAH or did not find any efficacy in the patients (Akdemir et al., 2009[6]; Dorhout Mees et al., 2012[33]; Wong et al., 2010[171]). Friedlich et al. (2009[42]) reported that MgSO_4_ at a dose of 0.6 g/hour has a prophylactic effect on cerebral vasospasm in the first 72 hours in a patient with ASAH. Overall, MgSO_4_ seems to be beneficial in the management of ASAH. 

#### MgSO_4_ and stroke

The use of MgSO_4_ 24 hours post-stroke shows a significant decrease in the infarct volume based on the findings of MRI (Kidwell et al., 2009[79]). Saver and colleagues performed a study on 1700 stroke patients in 2015 (Saver et al., 2015[132]). In their study, GCS, NIHSS and Barthel index were improved in the treatment group receiving MgSO_4_ compared with the control group (Veyna et al., 2002[158]). Singh et al. (2012[144]) showed neuroprotective properties of Mg in the stroke patients that received intravenous MgSO_4_ in comparison to the control group. Afshari and colleagues showed a significant effect of MgSO_4_ in decreasing the length of hospital stay in stroke patients (Afshari et al., 2013[2]). The significant effect of Mg on Barthel index, the length of hospital stays and recovery in 30 days post-stroke in the patients was reported by Lampl and colleagues (2001[88]). It was also suggested that one gram of MgSO_4_ daily decreases mortality rate in the non-cardiac ICU patients (Dabbagh et al., 2006[30]). Concurrent use of MgSO_4_ and nimesulide, and MgSO_4_ alone, has been reported to reduce the infarct volume in an animal model of stroke (Wang et al., 2012[163]; Zhu et al., 2004[175]).

Effect of MgSO_4_ on biomarkers in different neuropathies has been assessed in several studies (Bharosay et al., 2012[14]; Chan et al., 2005[23]; Friedlich et al., 2009[42]; Gao et al., 2013[45]; Gonzalez-Garcia et al., 2012[49]; Hassan et al., 2012[58]; James et al., 2009[69]; Johnson et al., 1993[72]; Lamers et al., 2003[87]; Mirrahimi et al., 2015[107]; Rahimi-Bashar et al., 2017[122]; Rinosl et al., 2013[125]). MgSO_4_ was shown to decrease S100B levels with little side effects (Hassan et al., 2012[58]). The increase of biomarkers like S100B and S-SNE has been reported with serum Mg levels below 1.2 mmol/L, and is associated with poor outcomes and a higher rate of mortality in patients with stroke (James et al., 2009[69]; Mirrahimi et al., 2015[107]). The decrease of these biomarkers may be correlated with an increase of Barthel index (James et al., 2009[69]). S100B has more sensitivity and specify than S-NSE (Gonzalez-Garcia et al., 2012[49]; Lamers et al., 2003[87]). Increase in S100B levels is associated with an increase in infarct size and NIH stroke score (Jauch et al., 2006[70]; Mizukoshi et al., 2013[108]). Increase in serum NSE levels has also been reported to be associated with an increase in post-stroke disability (Bharosay et al., 2012[14]). 

Gao and colleagues reported that 5 to 10 mmol/L of intravenous MgSO_4_ decreases inflammatory biomarkers such as nitric oxide, prostaglandin E2, interleukin 1β and tumor necrosis factor-α (Gao et al., 2013[45]). Concurrent use of neuroprotective agents and thrombolytic therapy is a promising treatment for acute ischemic stroke (Chen et al., 2002[26]; Ovbiagele et al., 2003[119]).

#### Mg and Traumatic brain injuries (TBI)

TBI is an important health problem with high a mortality and morbidity rate (Maas et al., 2008[95]). Studies on animal models have shown that Mg can increase the survival of neurons in cerebral ischemia and traumatic brain injury (Schanne et al., 1993[134]; Sirin et al., 1998[145]). 

Numerous studies have reported that Mg plays an important role in the prevention and treatment of central nervous system (CNS) injuries. Magnesium protects neurons from ischemic injuries and supports neuronal survival following TBI with different mechanisms such as: (1) blocking NMDA channels, (2) inhibition of presynaptic excitatory neurotransmitters, (3) inhibition of voltage-gated calcium channels, and (4) potentiation of presynaptic adenosine. Moreover, Mg can relax vascular smooth muscles and enhance cerebral blood flow. Serum total and ionized Mg levels are reduced after head injuries (McIntosh, 1993[103]; Memon et al., 2009[105]). The entrance of Mg into the CNS is dependent on the integrity of the BBB. In animal models, traumatic head injuries will facilitate entrance of Mg into the CNS for at least 24 hours (Habgood et al., 2007[56]; Heath and Vink, 1998[59]). The permeability of BBB in personal traumatic head injuries is not always present (Miller and D'Ambrosio, 2007[106]).

### MgSO_4_ in other patients admitted to the ICU

The beneficial effect of MgSO_4_ in ICU patients was described and assessed by researchers using different assessment methods (SOFA score, GCS, Rankin score, RASS score, APACHE score, NIH stroke scores, Barthel index, infarction volume, sepsis, tissue oxygenation index, mechanical ventilation and intubation requirement, length of hospital and ICU stay, and mortality) (Afshari et al., 2013[2]; Chen et al., 2015[25]; Dabbagh et al., 2006[30]; Jiang et al., 2017[71]; Kidwell et al., 2009[79]; Kumar et al., 2015[85]; Mousavi et al., 2010[110]; Muir and Lees, 1995[112]; Saver et al., 2015[132]; Singh et al., 2012[144]; Wang et al., 2012[163]; Zafar et al., 2014[172]; Zhao et al., 2016[174]; Zhu et al., 2004[175]).

The neuroprotective effect of MgSO_4_ in diffuse axonal injury has been shown by Zhao et al. (2016[174]). The intervention group in the referred study showed higher Glasgow coma scale (GCS) and lower serum neuron-specific enolase level (S-NSE), but the length of ICU stay and mortality did not differ between control and intervention groups (Habgood et al., 2007[56]). The presence of hypomagnesaemia in 374 ICU patients was reported by Chen and colleagues. Their results showed that hypomagnesaemia was correlated with increased length of ICU stay, SOFA score and mortality rate (Chen et al., 2015[25]). The mortality rate in the ICU patients with hypomagnesaemia was reported as 74 % in comparison with 36 % in patients with normal serum Mg levels (Zafar et al., 2014[172]). 

Hypomagnesaemia had a higher incidence in the alcoholic patients and patients with diabetes mellitus, sepsis, hepatic cirrhosis and chronic kidney disease. Higher need to mechanical ventilation, increase in the length of mechanical ventilation, increase in the risk of sepsis, higher APACHE score, decrease in NIHS score, decrease in serum albumin level and hypokalemia were also reported in these patients (Jiang et al., 2017[71]; Kumar et al., 2015[85]; Mousavi et al., 2004[109], 2010[110]; Muir and Lees, 1995[112]). MgSO_4_ cannot improve the strength of respiratory muscles in the critically ill patients under mechanical ventilation (Johnson et al., 1993[72]). Serum Mg level is a key factor determining the outcome of the patients in ICU (Rahimi-Bashar et al., 2017[122]). The normal level of serum Mg was associated with shorter time under mechanical ventilation and intubation and decreased ICU stay (Lampl et al., 2001[88]).

In patients admitted to the ICU after major abdominal surgery, serum Mg level should be checked daily because two-thirds of patients after abdominal surgery are diagnosed with hypomagnesaemia (Selvaraj and Syed, 2014[136]). As stated earlier, hypomagnesaemia is widely observed in the ICU, thus Mg replenishment should be considered in patients admitted to the ICU. For this reason, MgSO_4_ is an important drug in the ICU. MgSO_4_ can increase brain tissue oxygenation index by 34 % after cerebral artery occlusion (Chan et al., 2005[23]). 

Electrolyte imbalance following hypomagnesaemia has been reported by researchers in the ICU (Buckley et al., 2010[21]; Elin, 1994[37]; Faber et al., 1994[39]; Gonzalez et al., 2013[48]; Sedlacek et al., 2006[135]). Hypomagnesaemia can lead to a 2-3-fold increased mortality in ICU, and is one of the main causes of hypokalemia and hypocalcemia. It is also associated with hyponatremia and hypophosphatemia (Elin, 1994[37]; Gonzalez et al., 2013[48]; Sedlacek et al., 2006[135]). Mg has a major role in the transport of potassium, and simultaneous correction of hypomagnaesemia and hypokalemia is mandatory (Sedlacek et al., 2006[135]). Gupta et al. (2009[54]) showed that in a critically ill patient, administration of potassium and calcium is not sufficient to correct hypocalcemia and hypokalemia. Correction of hypomagnesaemia and control of Mg level in serum is highly recommended in patients with hypocalcemia and hypokalemia (Gupta et al., 2009[54]).

Magnesium sulfate was suggested by some authors to be efficacious in cardiac operations such as atrial fibrillation (AF), coronary artery bypass surgery and heart valve surgeries (Gu et al., 2012[53]; Lee et al., 2016[90]; Lip, 2016[93]; Mazurek and Lip, 2017[102]; Talkachova et al., 2016[150]; Treggiari-Venzi et al., 2000[154]). Low serum Mg level and older age have been reported as risk factors for AF (Treggiari-Venzi et al., 2000[154]). Atrial fibrillation is also one of the risk factors for ischemic stroke (Talkachova et al., 2016[150]). The role of MgSO_4_ for the management of AF is controversial. Use of intravenous MgSO_4_ without any other drugs in 16 patients was able to return heart rhythm to normal sinus rhythm after atrial fibrillation crisis (Sleeswijk et al., 2008[146]). Kaplan et al. reported that MgSO_4_ alone is not useful in the management of AF (Kaplan et al., 2003[76]). Concurrent use of MgSO_4_ with amiodarone in a post-operative patient with thorax surgery was reported to be beneficial for the prophylaxis against AF (Khalil et al., 2012[78]). In subjects with coronary bypass surgeries, MgSO_4_ was reported to reduce the risk of AF by 36 percent (Gu et al., 2012[53]). Administration of intravenous MgSO_4_, pre-operatively, post-operatively and during the heart valve surgery, decreased the risk of AF (Laiq et al., 2013[86]).

## Conclusion

Despite the controversial views on the effects of MgSO_4_ as a neuroprotective agent, current evidence suggests that MgSO_4_ is an important part of the management of ICU patients (Table 1(Tab. 1)). (References in Table 1: Kaplan et al., 2003[76]; Khalil et al., 2012[78]; Laiq et al., 2013[86]; Sleeswijk et al., 2008[146]; Treggiari-Venzi et al., 2000[154]; Akdemir et al., 2009[6]; Hassan et al., 2012[58]; Dorhout Mees et al., 2012[33]; Muroi et al., 2008[114]; van Norden et al., 2005[157]; Stippler et al., 2006[148]; van den Bergh et al., 2005[156]; Veyna et al., 2002[158]; Westermaier et al., 2010[166]; Wong et al., 2010[171]; Chan et al., 2005[23]; Zhao et al., 2016[174]; Afshari et al., 2013[2]; Lampl et al., 2001[88]; Muir et al., 2004[113]; Mirrahimi et al., 2015[107]; Muir and Lees, 1995[112]; Saver et al., 2015[132]; Singh et al., 2012[144]; Singh et al., 2008[143]; Boonyavorakul et al., 2000[16]; Green and Rothrock, 1992[51]; Silverman et al., 2002[141]; Porter et al., 2001[120]; Scarfone et al., 2000[133]; Ciarallo et al., 1996[29]; Ciarallo et al., 2000[28]; Gurkan et al., 1999[55]; Devi et al., 1997[32]; Bloch et al., 1995[15]; Tiffany et al., 1993[152].) Magnesium sulfate is essential to correct hypomagnesaemia and can decrease mortality rate, decrease the length of ICU stay, and is associated with reduced outcomes in patients admitted to the ICU. Because of the high prevalence of hypomagnesaemia and necessity of intravenous MgSO_4_ therapy in the ICU, serum Mg levels should be checked on a daily basis.

## Conflict of interests

The authors have no competing interests to declare.

## Figures and Tables

**Table 1:**
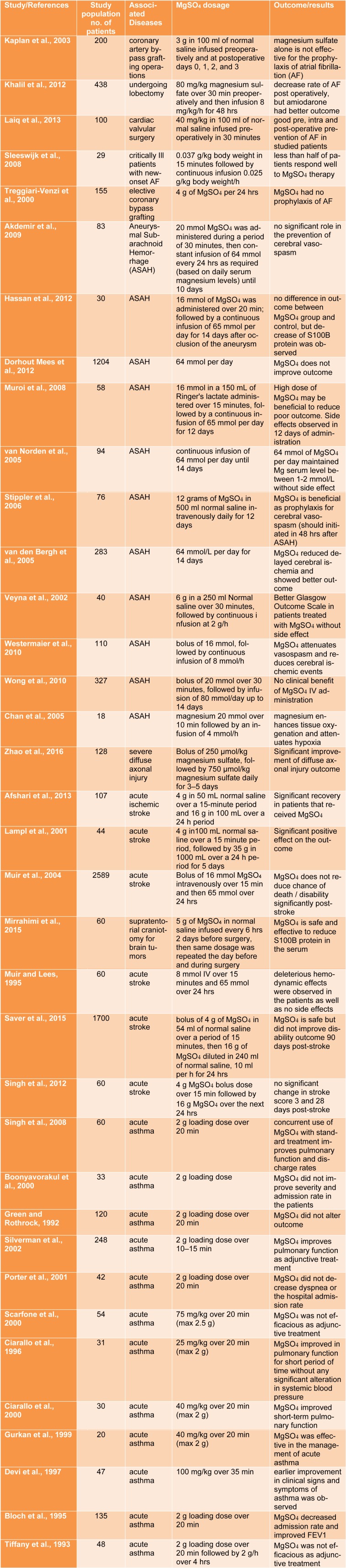
Summary of clinical studies evaluating the role of magnesium sulfate (MgSO_4_) in critically ill patients
